# Effect of temperature on the dynamic parameters of silty clay in a seasonally frozen region

**DOI:** 10.1038/s41598-023-40261-y

**Published:** 2023-08-12

**Authors:** Haotian Guo, Yuli Lin, Chao Sun, Xin Mao, Jinfeng Li

**Affiliations:** 1 School of Geometrics and Prospecting Engineering, Jilin Jianzhu University, Changchun, 130118 China; 2 College of Construction Engineering, Jilin University, Changchun, 130026 China

**Keywords:** Civil engineering, Geology

## Abstract

The effect of temperature on the dynamic parameters of silty clay in a seasonally frozen region was assessed using a GDS dynamic triaxial test system. The strength parameters, dynamic elastic modulus, damping ratio, and other dynamic parameters of the soil samples were analyzed under different temperature conditions. The results demonstrated that the shear strength parameters (internal friction angle and cohesion) of the silty clay under a dynamic load increased significantly with decreasing temperature, and the internal friction angle increased sharply below 0 °C. The dynamic elastic modulus increased as the temperature decreased and changed significantly in the ice-water phase change region. The slope of the dynamic stress–strain curve of the soil sample increased significantly with decreasing temperature. As the temperature decreased, the damping ratio reduced, and the ability of the soil to absorb seismic waves declines. The research results provide new data and information to guide construction projects in seasonally frozen region.

## Introduction

Frozen soil occurs widely globally and covers about 70% of the global land area^1^. Frozen soils can be classified into short-term frozen soils with a freezing time of a few hours to half a month, permafrost soils with a freezing time of two or more years, and seasonally frozen soils with a freezing time of half a month to a few months. Seasonally frozen soils and permafrost soils cover about 23% of the global land area^2^. Most of these soils are located in the middle and high latitudes in the northern and southern hemispheres, areas of extensive human activities, belong to the seasonally frozen region. Recent years due to the global warming caused by ecological changes, the soil in some multi-year frozen areas has thawed, resulting in an expansion of seasonally frozen soils. Therefore, it is crucial to study the mechanical parameters of soils in seasonally frozen regions to ensure sustainable construction.

Changes occurring in soils are more complex when dynamic loads rather than static loads are applied. Research on frozen soils and the number of engineering construction projects in seasonally frozen regions have increased. Analyzing the mechanical properties of seasonally frozen soils is particularly urgent, especially under dynamic loads. Most studies on frozen soil dynamics have focused on soil strength, the dynamic stress–strain relationship, the dynamic creep characteristics, the seismic response characteristics, the dynamic characteristics of pile foundations, and the dynamic response to train loads^3^. The dynamic parameters of soils in frozen regions are critical for the engineering design of high-rise buildings, bridges, ports, airports, and high-speed railroads and are indispensable for numerical simulations. Therefore, it is necessary to analyze the dynamic parameters of seasonally frozen soils.

Many experts and scholars have researched the dynamic properties of frozen soils in recent years, providing informative research results. Zhao et al.^4^ found that when a dynamic load was applied to frozen soil, the pores closed under pressure, increasing the strength and the dynamic elastic modulus. The pressure caused the dislocation of soil particles and the destructions of linkage, forming cracks, which weakened the soil and a reduced the dynamic elastic modulus. Zhu et al.^5,6^ conducted dynamic triaxial creep tests on frozen loess in Lanzhou under different circumferential pressures. They proposed a creep model and discussed the significance of the model parameters and the effects of changes in the parameter values. Wu et al.^7^ studied the mechanical properties of remodeled Lanzhou frozen loess using dynamic triaxial tests and investigated the dynamic properties of the frozen soil under seismic loading. Gidel et al.^8^ used a dynamic triaxial test apparatus to examine the dynamic response characteristics of coarse-grained soils under different static deflection stresses. They derived an empirical equation describing the relationship between the cumulative plastic strain, the stress magnitude, and the number of vibrations of the dynamic load. Zhou et al.^9^ established an intrinsic structure model to determine the effect of temperature and strain rate on the dynamic stress–strain of the soil. Vinson et al.^10^ conducted simulated seismic load tests on frozen sandy soils to assess the effect of temperature on the dynamic elastic modulus and damping ratio. Zhang et al.^11^ used a dynamic triaxial test device to study the effect of temperature on the dynamic parameters of silty clay. The results showed that a temperature change had a larger effect on the dynamic shear modulus and a smaller effect on the stress–strain and dynamic damping ratios below than above the freezing point Jiao et al.^12^ conducted tests on frozen soil at -1 °C and analyzed the hysteresis loops under dynamic loading. It was found that the area of the hysteresis loops increased with an increase in the maximum dynamic stress, indicating an increase in energy dissipation and more damage to the sample. Li et al.^13^ analyzed the relationship between the dynamic parameters of frozen soils and the influencing factors using low-temperature dynamic triaxial tests. Vision et al.^14,15^ studied the effect of freezing temperatures on pulverized soils and found that the dynamic elastic modulus was positively correlated with the water content at low freezing temperatures and negatively correlated at high freezing temperatures. Ling et al.^16^ concluded from low-temperature dynamic triaxial tests that the maximum dynamic shear modulus of frozen soils increased significantly with decreasing negative temperatures, increased with increasing pressure, and decreased with increased vibration frequency. The dynamic shear modulus ratio increased with an increase in the vibration frequency in the first loading stage^17^. Xu et al.^18^ conducted triaxial tests on frozen and unfrozen Genhe silty clay and found that the stress–strain curve of silty clay changed from softening to hardening as the confining pressure and temperature increased and the compaction decreased. Zhao et al.^19^ conducted a low-temperature cyclic dynamic triaxial test on frozen silt soil roadbeds on the Qinghai-Tibet Plateau. They analyzed the dynamic properties of the soil for different freezing temperatures, initial moisture contents, compaction degrees, and confining pressure conditions. It was observed that the dynamic shear modulus increased with decreasing freezing temperature, increasing moisture content, and increasing compaction degree and compaction pressure. Jia et al.^20^ studied the energy loss and the dynamic properties of frozen soils under radial dynamic loading using radial impact compression tests. They found that the dynamic mechanical properties of frozen soils subjected to radial dynamic loading were closely related to the loading strain rate and temperature. Qiu et al.^21^ analyzed carbonate saline soils in cold regions using indoor tests and other methods, considering the effects of different numbers of freeze–thaw cycles and the salt content on the shear strength. The test results showed that the shear strength of the carbonate saline soils and the number of freeze–thaw cycles were inversely correlated with the salt content. Lijith et al.^22^ investigated the effects of the volumetric ice content, shear rate, and vertical stress on the shear strength properties of frozen, fine sand using an improved, low-cost temperature-controlled direct shear chamber device. The experimental results showed that the cohesion, cut line modulus, and shear expansion angle increased with the shear rate and volumetric ice content. However, no monograph on frozen soil dynamics has been published, and research on frozen soils is in the early exploratory stage, especially the dynamics of seasonally frozen soils. Most studies have focused on specific soils, such as loess, and used remodeled soils, whereas relatively few studies investigated the dynamics parameters of widely distributed silty clay.

The literature review indicates that many scholars have analyzed the mechanical properties of frozen soils, whereas few studies focused on the shear strength parameters of silty clay under dynamic loads and different temperatures. Therefore, we used silty clay samples from a typical seasonally frozen region (Changchun, China) and a GDS dynamic triaxial test system to compare the dynamic parameters of soil samples at different temperatures and analyze the effect of temperature on the dynamic parameters of silty clay in a seasonally frozen region. The research results provide new data and information to guide construction projects in these regions.

## Basic properties of soil samples

**Figure 1:**
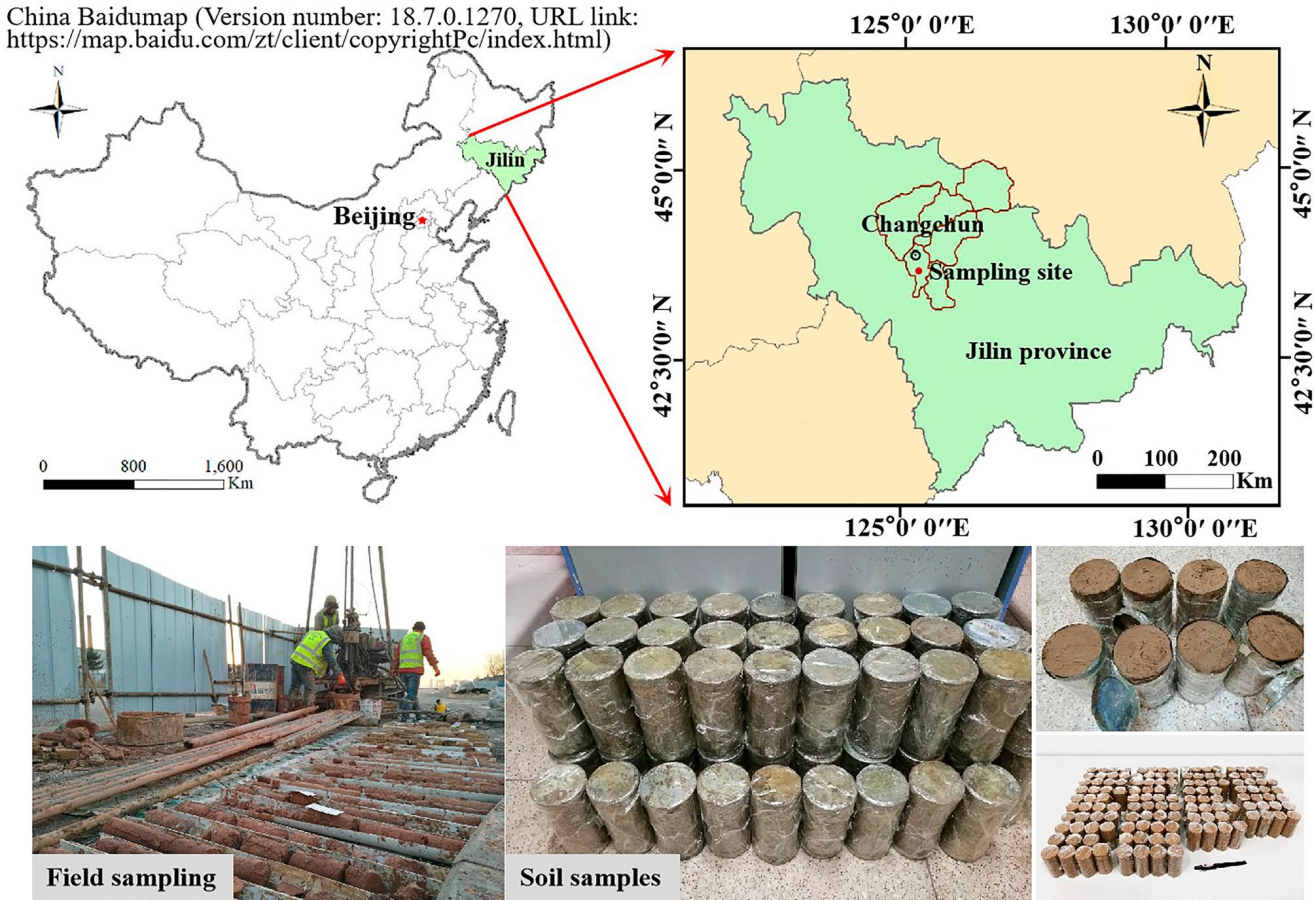
Sampling locations and soil samples.

**Figure 2:**
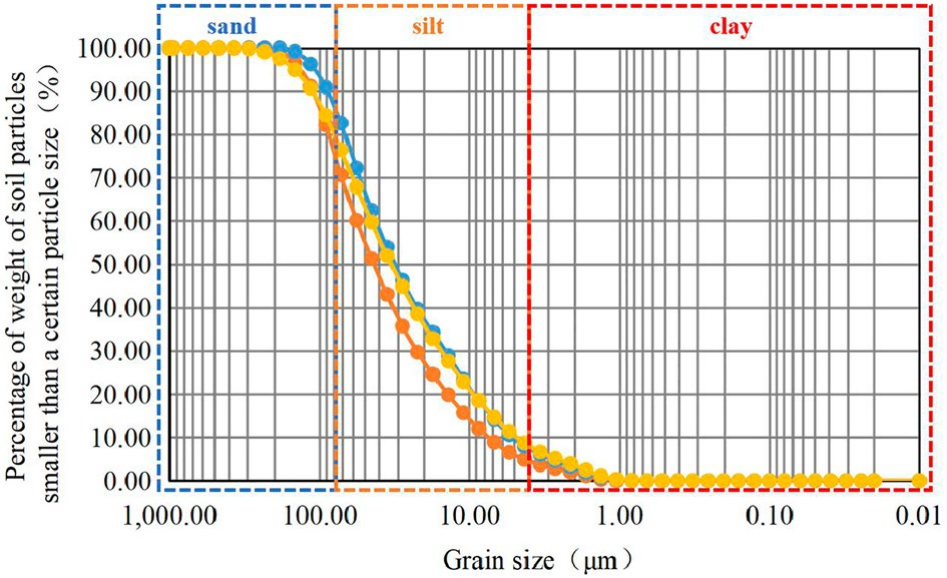
Particle size distribution of the soil.

**Figure 3:**
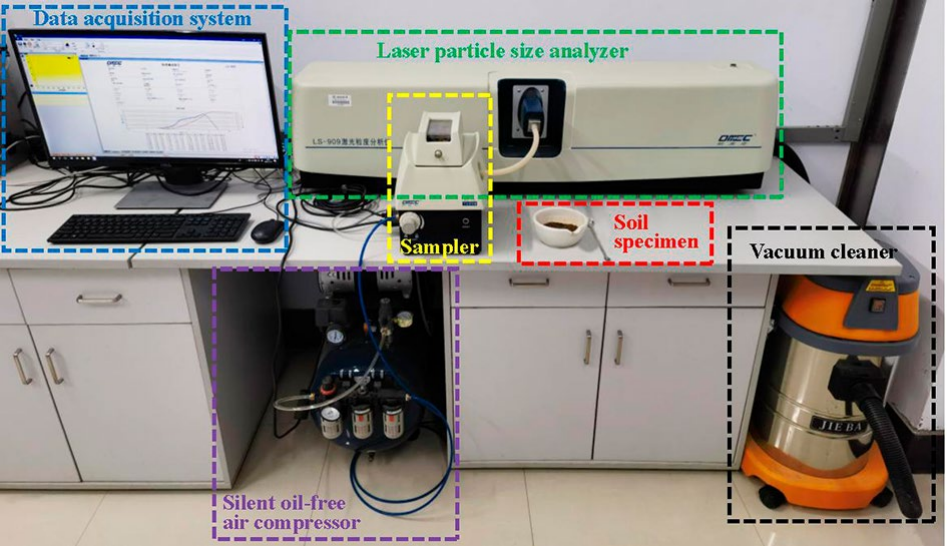
Laser particle size analyzer (BT-9300H).

The test samples were obtained from Jilin Avenue in Changchun, Jilin, China, a typical seasonally frozen region. The sampling site and the soil samples are shown in Fig. 1. According to the weather, geological and other related information of the sampling location, it can be seen that the average depth of frozen soils is 1.7 m. Excluding the effect of freezing and thawing on soil properties in the natural state, the sampling depth was selected as 4–7 m. The samples were sealed immediately after sampling and stored in the same vertical direction.Twelve soil samples of 10 cm diameter by 20 cm height were prepared into 36 samples of 38 mm × 76 mm columns , and particle size distribution, moisture content, compression coefficient, compression modulus, etc. of the soil samples were measured according to the ASTM standard^23–25^. The particle size distribution of the soil samples (Fig. 2) was obtained by a laser particle size analyzer (Fig. 3). The test results demonstrated that the content of clay particles with a particle size less than 0.005 mm ranged from 8.87% to 14.65%, with an average value of 13.17%. The content of silt particles with particle sizes from 0.005 mm to 0.075 mm ranged from 69.79% to 76.76%, with an average value of 73.30%. The uniformity coefficient C_U_ and the curvature coefficient C_C_ were calculated. C_U_ > 5 and C_C_ < 1, indicating that the soil sample was homogeneous and the soil was gap-graded. The soil samples contained predominantly silt and clay particles, and the content of the silt particles was high. The soils are identified according to the uscs system as CL.These soils have high capillary action, affecting their physical properties in freezing conditions^26^.

**Figure 4:**
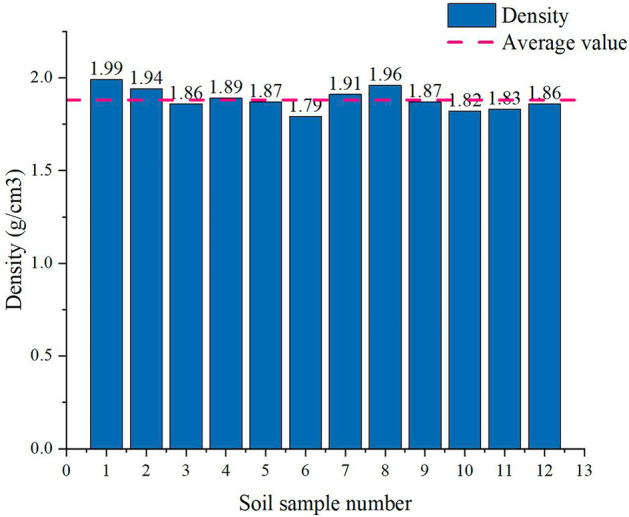
Density of soil samples.

**Figure 5:**
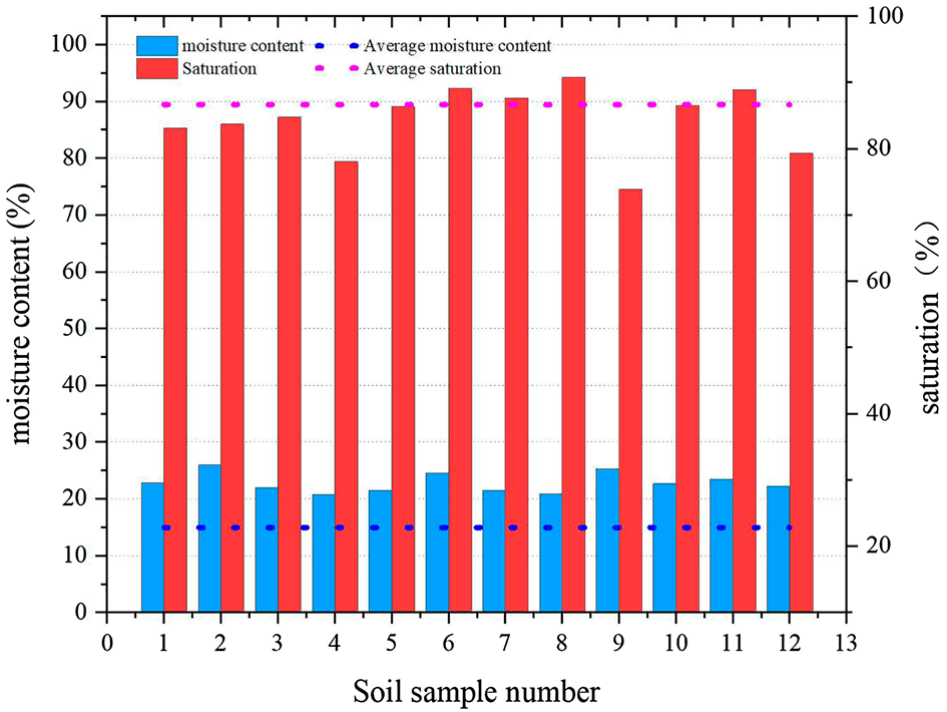
Moisture content and saturation of soil samples.

**Figure 6:**
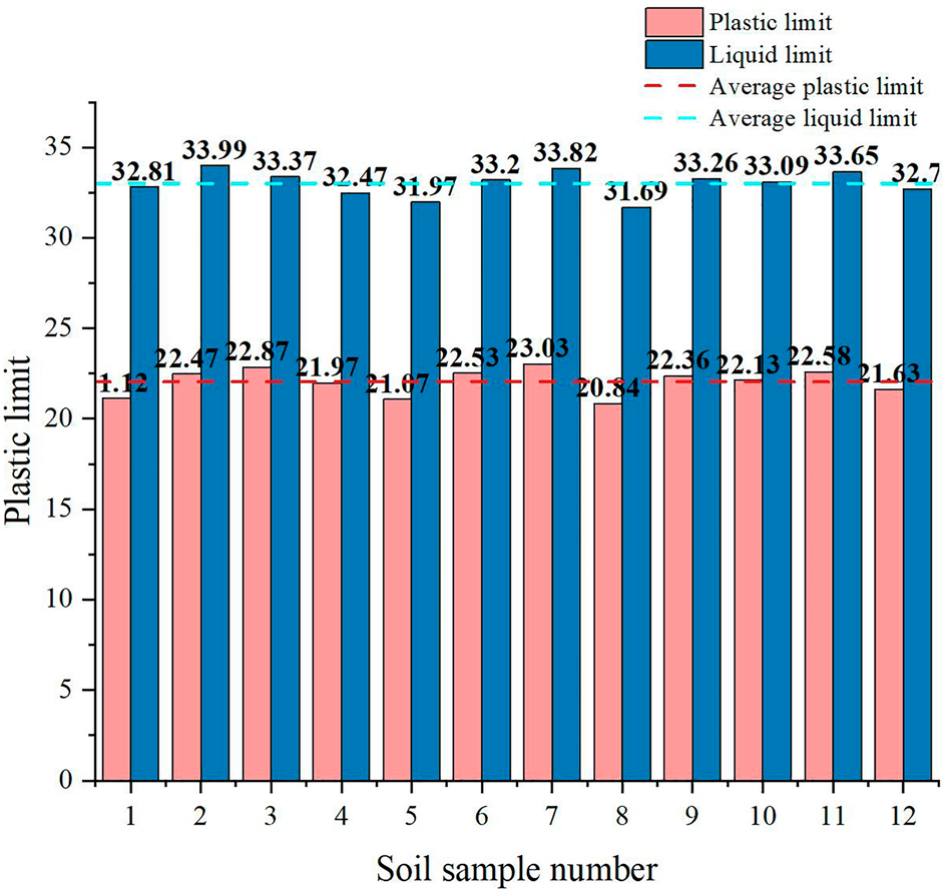
Liquid limit and plastic limit of soil samples.

**Figure 7:**
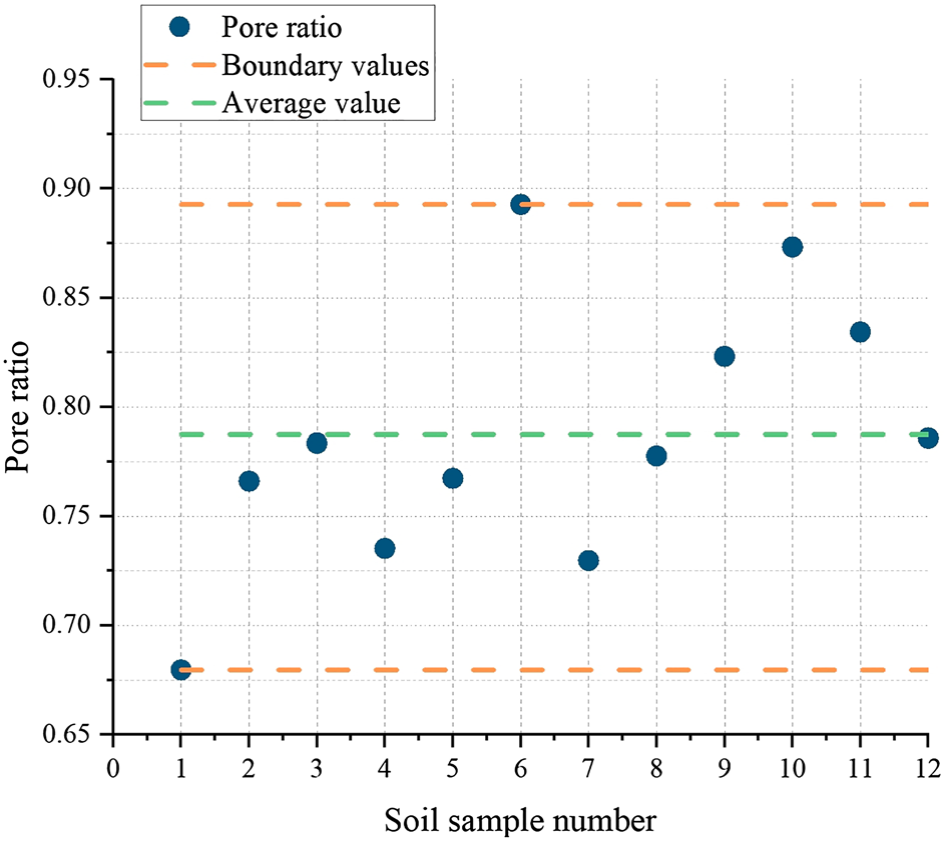
Porosity of soil samples.

The natural density measured by the ring knife method was 1.86 g/cm^3^-1.99 g/cm^3^, and the average wet density was 1.89 g/cm^3^. The moisture content measured by drying was 20.7–25.8%, and the average moisture content was 22.8%. The liquid limit range was 31.73–34.06%, with an average liquid limit of 33.05%, and the plastic limit range was 20.85% ~ 23.07%, with an average plastic limit of 22.06%. It was measured using a soil liquid-plastic limit test instrument. The pore ratio was converted from 0.64 to 0.90 using a conversion equation, and the average pore ratio was 0.79. The compressive modulus was measured using an automatic consolidation test system. It was 4.82 MPa to 5.7 MPa, and the compression coefficient ranged from 0.28 MPa^−1^ to 0.40 MPa^−1^. The density, water content, liquid-plastic limit, pore ratio, and the compression modulus and compression coefficient are shown in Figs. 4, 5, 6 and 7 (shows in the Supplementary Fig. S1), respectively. This indicates that the soil samples are moderately compressive clays in their natural state.

## Test equipment and test program

### Test instruments and test principles

The test used the GDS dynamic triaxial test system, which can simultaneously provide and control dynamic load, axial pressure, confining pressure, backpressure and temperature, and is characterised by relatively high accuracy in terms of temperature (− 20 °C ± 0.01 °C ~ 60 °C ± 0.01 °C), displacement (90mm ± 0.0008mm), and so on, consisting of a controller, pore water pressure sensor, data acquisition system, and GDSLab control system, as shown in Supplementary Fig. S2. The GDSLab control system was used to control the dynamic load, axial pressure, confining pressure, counter pressure, and temperature. The sensors transmit the measured data to the data acquisition system and the computer. The test included four stages: backpressure saturation, consolidation, cooling, and dynamic loading. In the backpressure saturation stage, the confining pressure is 20 kPa larger than the backpressure. A loading plate is controlled by the software to ensure that the soil is saturated. The backpressure saturation stage is completed when the drainage volume or pore pressure is equal to the backpressure and remains stable. Isotropic consolidation is performed in the consolidation stage. The soil specimen has been consolidated when the drainage rate is less than 0.1 cm^3^ per h. In the cooling stage, the temperature is lowered to the target temperature by circulating glycol antifreeze (freezing point − 45 °C) in the wall of the outer pressure chamber. The dynamic loading stage consists of graded loading. In order to simulate seismic loading,undrained conditions were used due to the transient nature of earthquakes.

### Test program

The boundary between high-temperature and low-temperature frozen regions is − 1 °C, i.e., in low-temperature frozen regions, T < − 1 °C, and in high-temperature frozen regions, − 1 °C ≤ T ≤ 0 °C^27^. The average winter temperature in Changchun is estimated to be − 15 °C. A temperature change affects the dynamic properties of high-temperature frozen soils more at temperatures above − 4 °C. Therefore, we used − 15 °C, − 4 °C, and − 1 °C as the initial test temperatures and − 20 °C and − 10 °C as the subsequent test temperatures. Seasonally frozen soil is highly sensitive to temperature changes due to its unique structure. We used room temperature for comparison in the dynamic strength parameter test. The final test temperatures were the room temperature as a control group (25°C) and temperatures of − 1 °C, − 4 °C, − 10 °C, − 15 °C, and − 20 °C.

The consolidation stress ratio of the soil is related to soil development, soil type, and other factors. The value of K_c_ is generally 0.25–2.5, and that of compacted fill is generally 0.7–1.25. In isotropic consolidation, K_c_ = 1.0, and the consolidation is completed when the drainage rate of the specimen is less than 0.1 cm^3^ per h. Based on the burial depth of the samples, confining pressures of 100 kPa, 200 kPa, and 300 kPa were used. Studies have shown that the seismic frequency (the frequency at which the energy density of seismic waves is the greatest) is typically 1.40 to 7.25 Hz^28^, and the fundamental frequency is approximately 1.0 Hz; thus, we used a frequency of 1 Hz. Seed et al. analyzed large amounts of seismic data^29^ and found that 65% of the maximum load amplitude was the equivalent amplitude of the cyclic load. Therefore, the load amplitude of this test was ${\tau }_{e}=$ 0.65 ${\tau }_{max}$.

Graded loading was used, and σ_min_ remained constant. The dynamic stress amplitude σ_m_ increased with the number of loading steps. The schematic diagram of the triaxial loading test is shown in Supplementary Fig. S3, and the loading amplitude at different levels is defined in Eq. (1).

1 $$ \sigma (t) = \sigma_{0} + \sigma_{m} \sin (2\pi ft), $$

where $\sigma_{0}$ is the initial static stress,$\sigma_{m}$ is the dynamic stress amplitude,$\sigma_{m} = \frac{1}{2}\left( {\sigma_{\max } - \sigma_{\min } } \right)$,$\sigma_{\max }$ is the maximum stress, $\sigma_{\min }$ is the minimum stress, $f$ is the loading frequency, and $t$ is the time.

The consolidated undrained test was performed according to the ASTM standard^30^ (Standard Test Method for Unconsolidated-Undrained Triaxial Compression Test on Cohesive Soils), and the soil sample was vacuum-saturated before the test. For soft clay soils, the end of the test occurs when the strain criterion reaches a specific value. When K_c_ = 1.0, the failure strain occurs when two times the amplitude of the axial dynamic strain 2*ε*_*d*_ reaches 5% or 10%. In this test, the termination condition was 5%.

### Test process

Before the test, we saturated the soil samples. They were placed into a vacuum saturator with a three plate mold and injected with distilled water after being in a vacuum state for two hours. Subsequently, the ventilation valve was opened to restore the atmospheric pressure, and the samples remained in this condition for more than 12 h. Since the soil was dense, the rest time was extended to 24 h-48 h under the condition of sufficient vacuuming.

The saturated soil sample was placed in the pressure chamber filled with antifreeze to cool the soil sample and apply a confining pressure to it. The backpressure and the confining pressure, which was 20 kPa higher than the backpressure, was applied. The backpressure saturation was completed when the drainage volume or the pore pressure was equal to the confining pressure and remained stable. Due to the small pore size of silty clay, a B-value greater than 0.95 is difficult to achieve during multistage backpressure saturation. The consolidation time of the test was 12 h. The target temperature was set by the advanced loading module, and the specimen was cooled using antifreeze circulation. The pressure chamber was covered with cling film the preserve heat.

## Test results and discussion

### Effect of temperature on soil dynamic strength parameters

In an undrained test, the dynamic stress on the damage surface is borne by the pore water^29^ and does not cause soil compaction. Therefore, the dynamic strength of saturated soil is related to the static positive stress *σ*_*s*_ on the damage surface. The amplitude *σ*_*d*_ corresponding to the vibration time is superimposed on *σ*_1_, i.e., *σ*_*1d*_ = *σ*_*1*_ + *σ*_*d*_ and the small principal stress *σ*_*3d*_ = *σ*_*3*_, and Mohr’s stress circle has a diameter of *σ*_*1*_ + *σ*_*d*_-*σ*_*3d*_. The intercept and slope of the envelope tangent line correspond to the dynamic cohesion and dynamic internal friction angle, respectively. Mohr’s stress circles at different temperatures for confining pressures of 100 kPa, 200 kPa, and 300 kPa with an amplitude of ${\tau }_{e}=$ 0.65 ${\tau }_{max}$ are shown in Supplementary Fig. S4. The dynamic cohesion and dynamic internal friction angle of the soil are shown in Supplementary Fig. S5. The blue lines in Fig. S5 show the trends of the cohesion at − 20 °C, − 15 °C, − 10 °C, − 4 °C, − 1 °C, and at room temperature. The cohesion increases with decreasing temperature and increases sharply in the ice-water phase change region near 0 °C. The frozen soil consists of unfrozen water, ice crystals, soil particles, gas, and other multi-phase media. When a dynamic load is applied to the medium with four phases, the movement, migration, diffusion, and phase change affect the soil’s physical properties. When the temperature is near 0 °C in the ice-water phase change region, the ice crystal content in the soil and the bond strength between the soil particles increase. The internal structure of the ice crystals changes, and the hydrogen ion activity decreases as the temperature decreases^31^. The cementation capacity of the soil increases as the temperature approaches 0 °C, causing a sharp increase in soil cohesion. In contrast, when the temperature is lower than the ice-water phase change zone, the rate of increase in the ice crystal particles is low, and the main factor affecting the soil cohesion is the hydrogen ion activity.

The effect of the temperature on the internal friction angle is shown in Fig. S5. The internal friction angle increases as the temperature decreases. It increases slowly when the temperature is higher than 0 °C and sharply when the temperature is lower than 0 °C. The unfrozen water in the soil was used as a lubricant to the particles. When the temperature is higher than the freezing temperature, the degree of water–ice phase transition is small and the unfrozen water content is high, the lubrication between the soil particles is relatively large.At this time, the occlusal force between the particles is poor, the angle of internal friction is small. As the temperature falls below 0 °C, the unfrozen water content decreases sharply. The pores between the soil particles are filled by ice crystals, and the force required to move the soil particles increases suddenly. The internal friction angle increases sharply, and the rate of change decreases as the temperature decreases.

### Effect of temperature on dynamic stress and strain

An analysis of the dynamic stress–strain curve is necessary to assess the dynamic properties of frozen soils. The dynamic stress–strain curve can be used to determine three soil properties: hysteresis, strain accumulation, and nonlinearity. Hysteresis refers to a lag time in the onset of the dynamic strain compared to the dynamic stress. A hysteresis curve is used to analyze hysteresis. Supplementary Fig. S6 shows the relationship between the dynamic shear stressτ and the dynamic shear strainγin a cycle. The nonlinearity of the dynamic strain is reflected by the backbone curve, which describes the relationship between the maximum dynamic shear stress $\pm \tau_{m}$ and the maximum dynamic shear strain $\pm \gamma_{m}$ of the soil under cyclic loading. The hysteresis curve is shown in Supplementary Fig. S7.

The dynamic stress–strain relationship at different negative temperatures in Supplementary Fig. S8 shows that the lower the temperature, the greater the dynamic stress required to reach the same strain. The dynamic stresses required to achieve 5% dynamic strain at different temperatures are as follows: 0.911 MPa at − 1 °C, 1.14 MPa at − 4 °C, 1.56 MPa at − 10 °C, 2.42 MPa at − 15 °C, and 3.053 MPa at − 20 °C. The reason for the difference is that a lower temperature causes the unfrozen water in the soil to freeze into ice crystals. The internal structure is denser since solid ice crystals are more rigid than liquid water. Therefore, the lower the temperature, the higher the dynamic stress required to reach the same strain.

### Effect of temperature on dynamic modulus of elasticity

The dynamic modulus of elasticity indicates the degree of stiffness softening of the soil under cyclic loading, i.e., the ability of the soil to resist deformation. In the elasticity theory, ${E}_{d}$ denotes the dynamic stress–strain relationship when the soil is subjected to cyclic loading, expressed as ${E}_{d}={\sigma }_{d}/{\varepsilon }_{d}$. In other words, the dynamic modulus of elasticity of the soil is equal to the slope corresponding to the fitted curve obtained from the Hardin-Drnevich hyperbolic equation^32^ (Eqs. (2) to (4)). The relationship curve for 1/${\sigma }_{d}/{\varepsilon }_{d}$ at different temperatures was plotted and fitted using the Hardin-Drnevich model (Supplementary Fig. S9):

2 $$ \sigma_{d} = \frac{{\varepsilon_{d} }}{{a + b\varepsilon_{d} }}, $$

3 $$ E_{d} = \frac{{\sigma_{d} }}{{\varepsilon_{d} }} = \frac{1}{{a + b\varepsilon_{d} }}, $$

4 $$ \frac{1}{{E_{d} }} = \frac{{\varepsilon_{d} }}{{\sigma_{d} }} = a + b\varepsilon_{d} , $$

where ${\varepsilon }_{d}$ is the dynamic strain amplitude,${\sigma }_{d}$ is the dynamic stress amplitude, and a and b are test parameters. When the dynamic strain tends to 0, a tends to 1/${E}_{d}$; when the dynamic strain ${\varepsilon }_{d}$ tends to infinity, b is the inverse of ${\sigma }_{d}$.

The fitting results of the test data at different negative temperatures are summarized in Supplementary Table S1. The R^2^ values are all greater than 0.98, indicating that the Hardin-Drnevich model provided a good fit for the dynamic stress–strain data. The test parameters a and b are significantly influenced by the temperature, and both increase with the temperature.

Supplementary Fig. S9 shows that the dynamic elastic modulus increases with a decrease in the temperature. The slope increases, and the dynamic elastic modulus exhibits the largest rate of change when the temperature is in the ice-water phase change region. The reason is that the unfrozen water content decreases, and the cementing capacity of the soil increases as the temperature decreases, increasing the dynamic elastic modulus. The results demonstrate that the temperature significantly affects the dynamic elastic modulus.

### Effect of temperature on damping ratio

During an earthquake, the foundation soil has a damping effect on the propagation of seismic waves. Damping refers to converting dynamic energy into heat or other energy, resulting in energy loss due to internal friction when the soil is subjected to dynamic action. The degree of energy absorption by the soil is reflected by the magnitude of the damping ratio λ, a critical parameter in the ground vibration response of the soil. λ is calculated as follows:

5 $$ \lambda = \frac{c}{{c_{cr} }} = \frac{c}{2m\omega } = \frac{1}{4\pi }\psi = \frac{1}{2\pi }\delta $$

6 $$ \psi = \frac{\Delta W}{W}. $$

Therefore,

7 $$ \lambda = \frac{1}{4\pi }\frac{\Delta W}{W}, $$

where $\Delta W$ is the energy lost by the soil in one cycle, and $W$ denotes the total energy.

The dynamic stress–strain data at different levels of dynamic loading are plotted to obtain the hysteresis curve. Since frozen soil is not an ideal viscoelastic material, the hysteresis curve is not closed under multistage loading but has an elliptical shape. The first and last data points are connected to close the curve. The damping ratio is calculated using Eq. (8):

8 $$ \lambda = \frac{1}{4\pi }\frac{{A_{0} }}{{A_{r} }} $$

where $A_{0}$ refers to the area of the region enclosed by the hysteresis curve shows in Supplementary Fig. S10, which is approximately equal to the energy loss $\Delta W$. $A_{r}$ is the area of the triangle $OAA^{\prime}$, which is equal to the maximum elastic strain energy stored in the soil.

The relationship between the damping ratio and the dynamic shear strain at different negative temperatures is shown in Supplementary Fig. S11. The damping ratio increases with an increase in the dynamic shear strain, and the rate of increase rises due to the increasing shear strain and loss or energy, increasing the damping ratio. At the same dynamic strain, the lower the temperature of the soil sample, the lower the damping ratio. The lower temperature causes the water inside the soil to freeze into ice crystals. The hardness and strength of the soil increase, the ability of the soil to absorb seismic waves is weakened, and the energy lost by the soil decreases, reducing the damping ratio.

## Conclusion

Dynamic triaxial tests were conducted to evaluate the dynamic strength parameters, stress–strain relationship, modulus of elasticity, and damping ratio of silty clay in a seasonally frozen region under different temperatures. The following conclusions were drawn.

1. As the temperature decreased and the unfrozen water in the soil froze after reaching the ice-water phase transition zone, the soil’s cementation degree, cohesion, and internal friction angle increased. The internal friction angle increased more slowly at temperatures higher than 0 °C and more sharply at temperatures lower than 0 °C.
2. As the temperature decreased, the ice crystal content and stiffness of the soil increased. The soil pores became denser, increasing the slope of the dynamic stress-strain curve of the soil as the temperature decreased. Thus, the stress required to reach the same strain increased.
3. The maximum cementation capacity of the soil occurred in the ice-water phase transition zone. The dynamic elastic modulus increased as the temperature decreased, and the fitted parameters a and b decreased. The fitted curve became steeper, and this effect was more pronounced in the ice-water phase change region.
4. The damping ratio λ was used to reflect the absorption capacity of the soil for seismic waves. As the temperature decreased, the unfrozen water in the soil froze. The stiffness of the soil increased, reducing its ability to absorb seismic waves. The rate of the loss of seismic energy decreased, and the damping ratio decreased with the temperature.

### Informed consent statement

The people in Fig. 1 are not the authors themselves, they are the sampler personnel, all of whom gave informed consent for the release of identifying information/images in the online open access publication.

### Supplementary Information

Supplementary Information.

## Data Availability

The data that supports the findings of this study are true and available, and are authorized by all the authors. Please contact the corresponding author if you would like to access the study data.
