# Supplementary material for: Effect of temperature on the dynamic parameters of silty clay in a seasonally frozen region
Source: Sci Rep. 2023 Aug 12;13:13141. doi: 10.1038/s41598-023-40261-y (PMC10423233; doi:10.1038/s41598-023-40261-y)
Supplement: Supplementary file 1 — Supplementary Information. [file 41598_2023_40261_MOESM1_ESM.pdf]

SUPPLEMENTARY INFORMATION

Effect of temperature on the dynamic parameters of silty clay in a seasonally frozen region

Haotian Guo[1,*], Yuli Lin[1], Chao Sun[1] , Xin Mao[1] , and Jinfeng Li[2]

[1] School of Geometrics and Prospecting Engineering, Jilin Jianzhu University, Changchun 130118, China

[2] College of Construction Engineering, Jilin University, Changchun 130026, China

*Corresponding author: Haotian Guo, e-mail: guohaotian@163.com

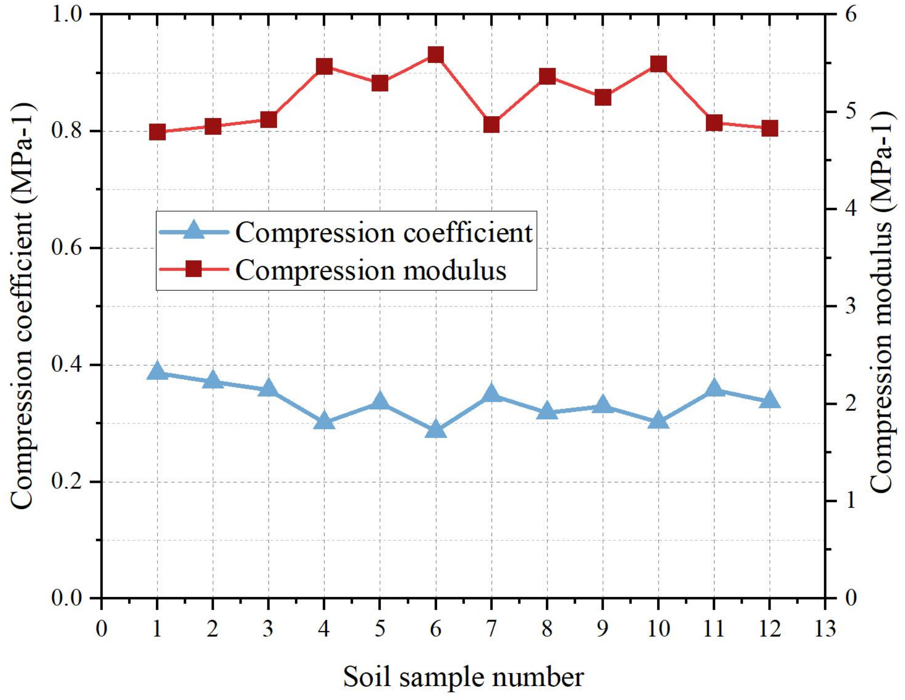

Supplementary Fig.S1 Compression coefficient and compression modulus of soil samples

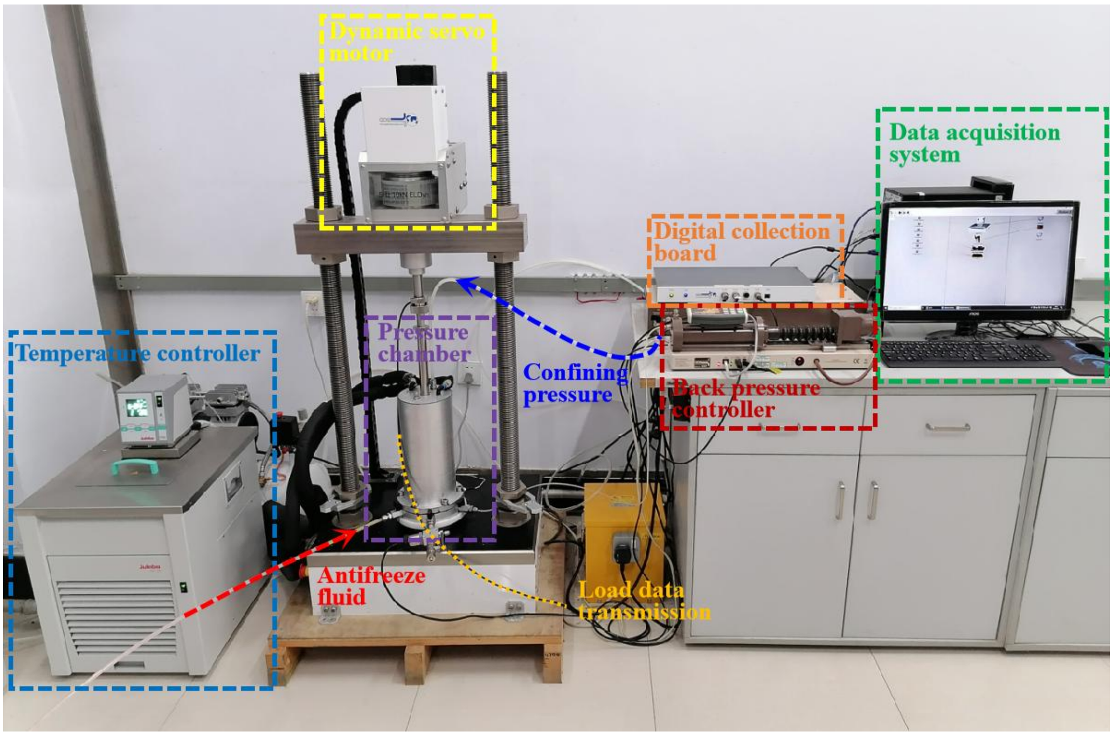

Supplementary Fig.S2 ELDyn dynamic triaxial system

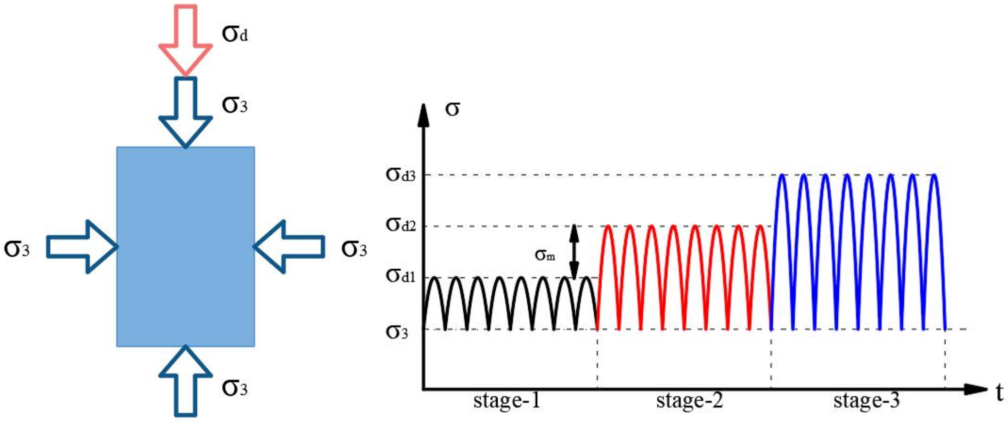

Supplementary Fig.S3 Schematic diagram of axial multistage cycle load loading

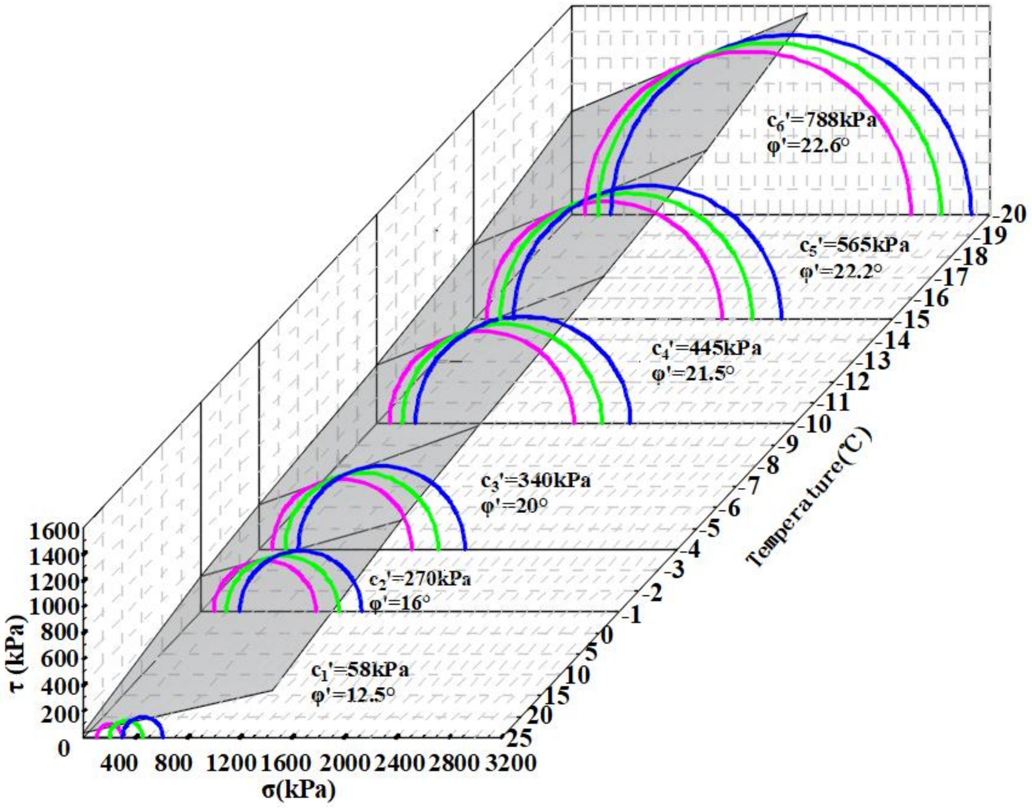

Supplementary Fig.S4 Mohr's stress circle at different temperatures

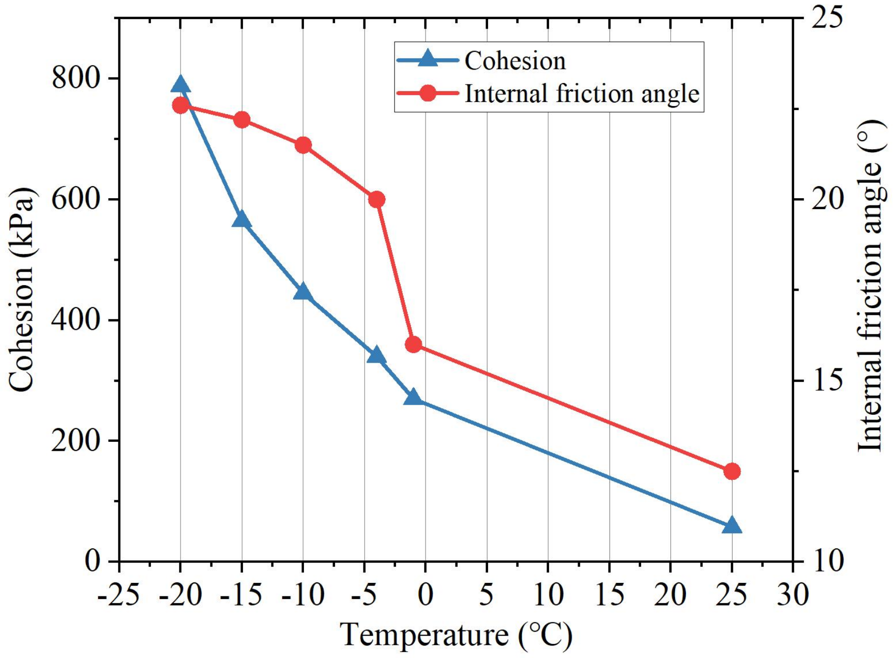

Supplementary Fig.S5 Plot of soil cohesion changes with temperature

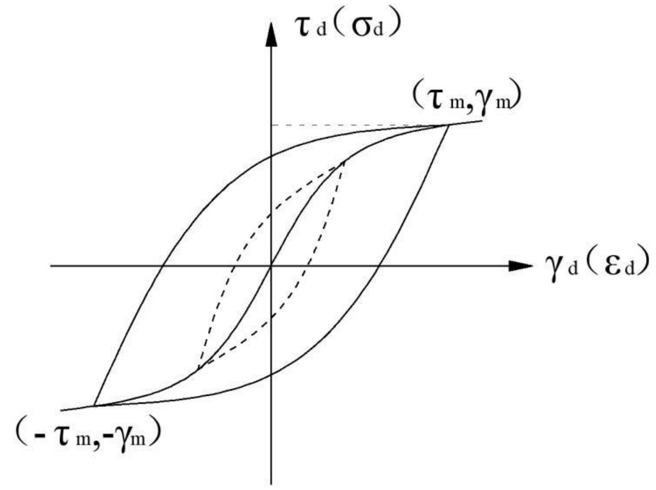

Supplementary Fig.S6 hysteresis loop

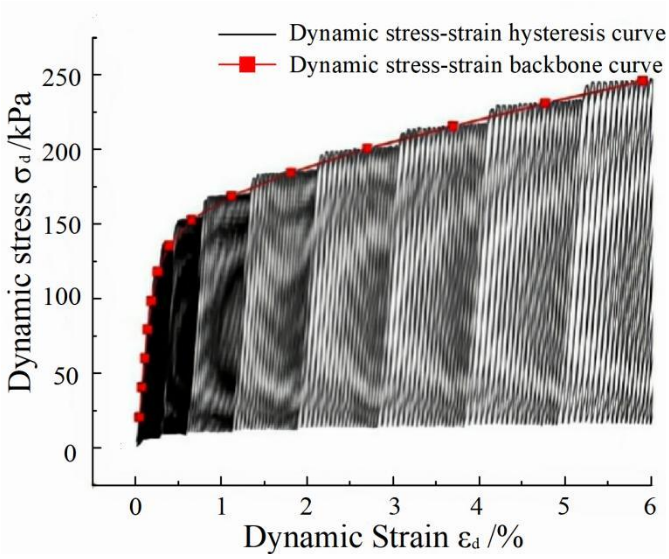

Supplementary Fig.S7 backbone curve

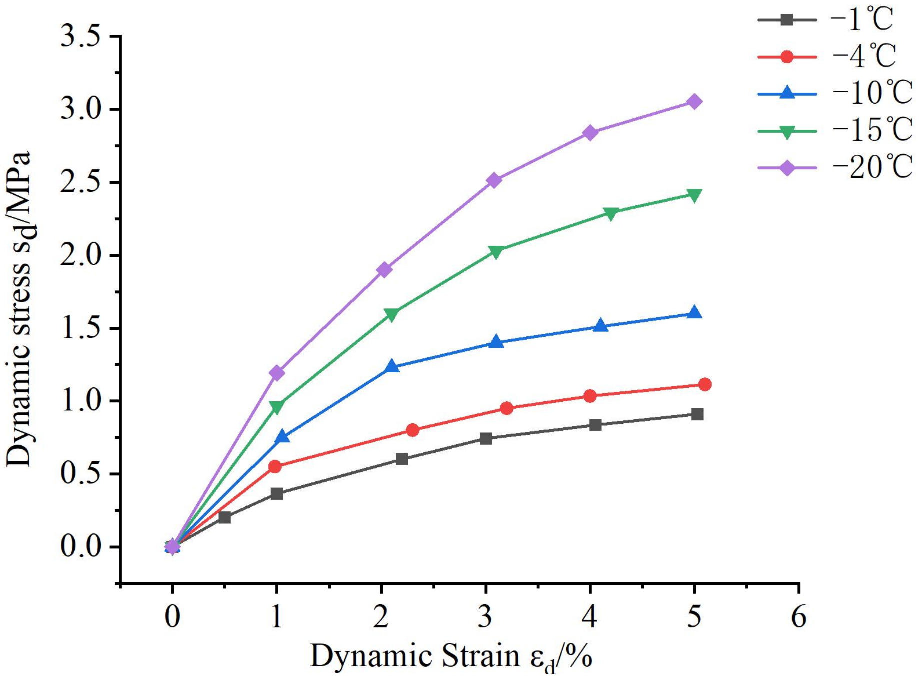

Supplementary Fig.S8 Dynamic stress-strain relationship at different temperatures

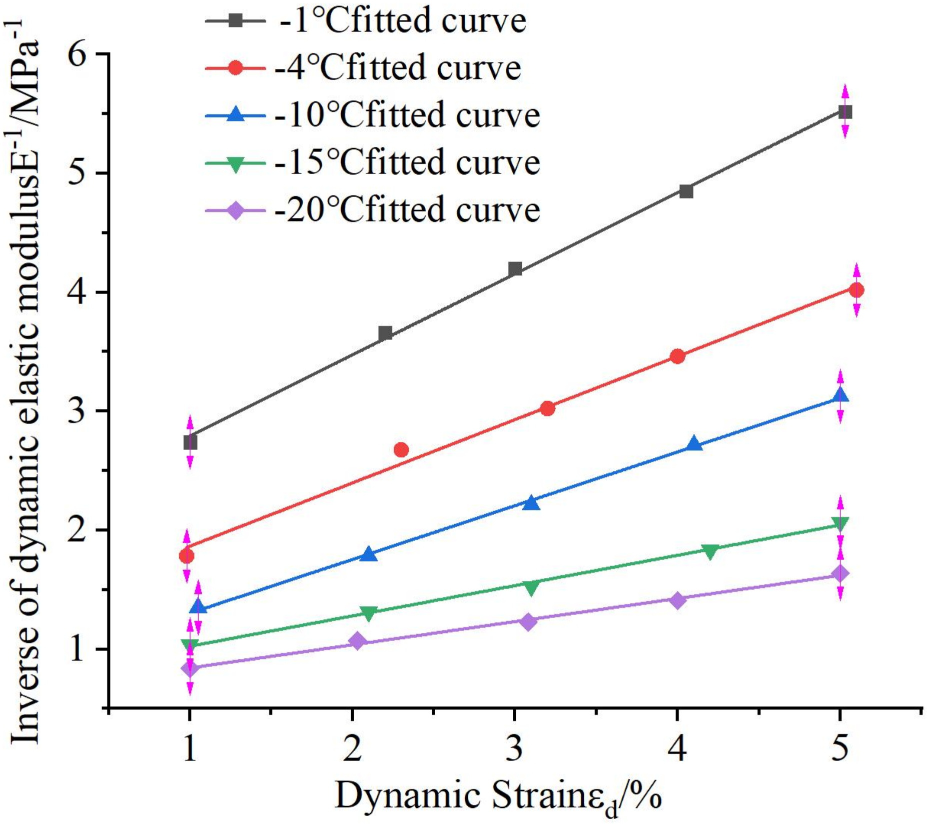

Supplementary Fig.S9 Fitting results at different temperatures for the H-D model

Supplementary Table S1 Fit results of the test data under the temperature influence

Test temperature（℃）	a	b	R^2
-1	2.110	0.682	0.998
-4	1.334	0.532	0.994
-10	0.850	0.452	0.998
-15	0.770	0.255	0.997
-20	0.648	0.194	0.995

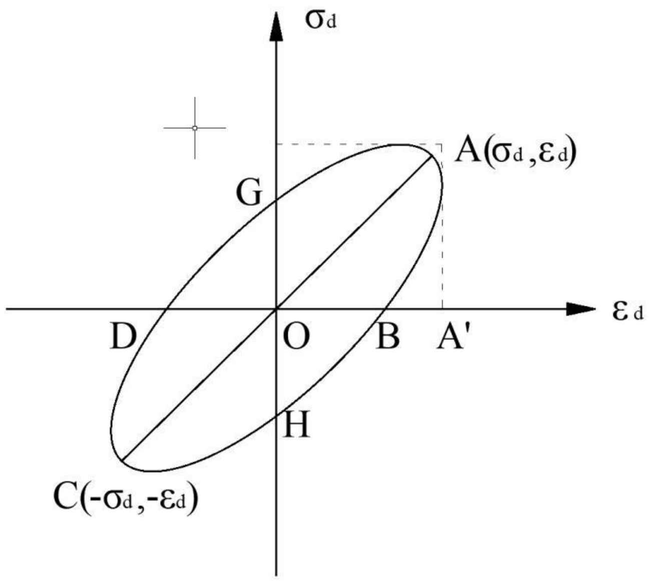

Supplementary Fig.S10 Delresis curve of ideal viscoelastomers

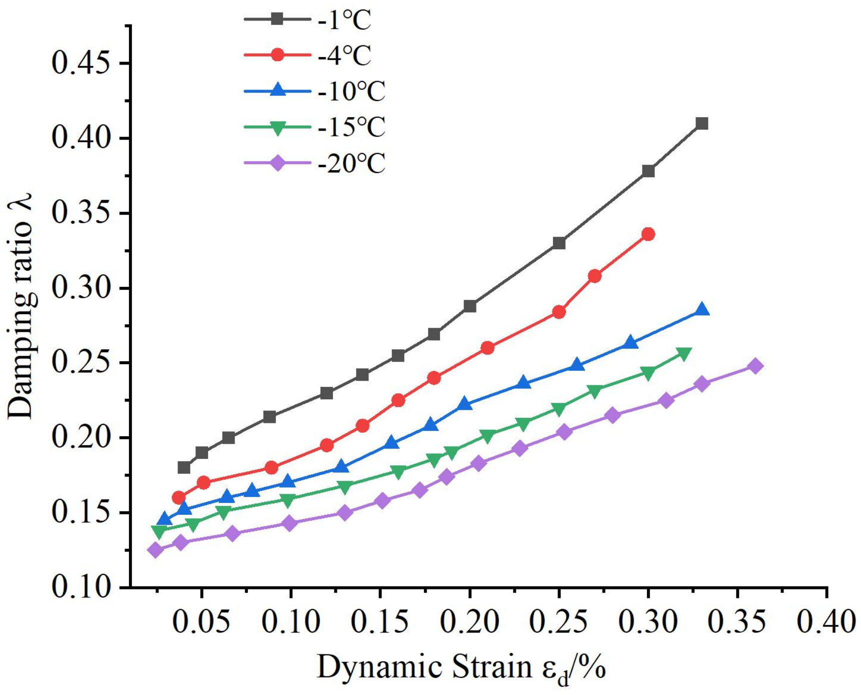

Supplementary Fig.S11 Damp ratio and dynamic shear should variable amplitude relationship at different temperatures
